# A Rare Case of the Digenic Inheritance of Long QT Syndrome Type 2 and Type 6

**DOI:** 10.1155/2019/1384139

**Published:** 2019-06-20

**Authors:** Annejet Heida, Lisette J. M. E. van der Does, Ahmed A. Y. Ragab, Natasja M. S. de Groot

**Affiliations:** Department of Cardiology, Erasmus Medical Center, Rotterdam, Netherlands

## Abstract

We report a 37-year-old woman with an out-of-hospital cardiac arrest caused by ventricular fibrillation due to digenic inheritance of long QT syndrome type 2 (*KCNH2* gene) and type 6 (*KCNE2* gene). During hospitalization, prolonged QTc intervals and frequent episodes of ventricular tachyarrhythmias manifested. Genetic testing identified a mutation of the *KCNH2* gene and an unclassified variant, most likely pathogenic, of the *KCNE2* gene. This digenic inheritance is extremely rare.

## 1. Introduction

In the past decennia, our knowledge of the genetic etiology of cardiac arrhythmias has enormously increased. Cardiac tachyarrhythmias may be caused by several hereditary diseases including primary electrical disorders, such as long QT syndrome (LQTS) [1]. The LQTS is characterized by a prolongation of cardiac repolarization (QTc >450 ms in men and >460 ms in women), leading to ventricular tachyarrhythmias such as torsade des pointes (TdP). Consequently, affected patients have an increased risk of syncope and sudden cardiac death (SCD). The genes that are associated with LQTS include *KCNQ1* (LQT1), *KCNH2* (LQT2), *SCNA5* (LQT3), *KCNE1* (LQT5), and *KCNE2* (LQT6) in addition to a few less frequent subtypes [2]. The most frequent variants of LQTS involve the *KCNQ1*, the *KCNH2*, and the *SCN5A* genes which account for approximately 90% of genotype-positive patients. Less frequent variants of LQTS involve the *KCNE1* and *KCNE2* genes [3, 4]. This case report is to our knowledge the first to describe the digenic inheritance of long QT syndrome type 2 (*KCNH2* gene) and type 6 (*KCNE2* gene).

## 2. Case Report

A 37-year-old Iraqi woman, living in the Netherlands, with no cardiac history, was brought to the emergency department with an out-of-hospital cardiac arrest (OHCA) due to ventricular fibrillation (VF). That evening, she had suddenly collapsed without any warnings. After two to three minutes, the police started with basic life support, and the paramedics arrived after ten minutes. On arrival, VF was documented, and after three direct current shocks, sinus rhythm was restored.

**Figure 1:**
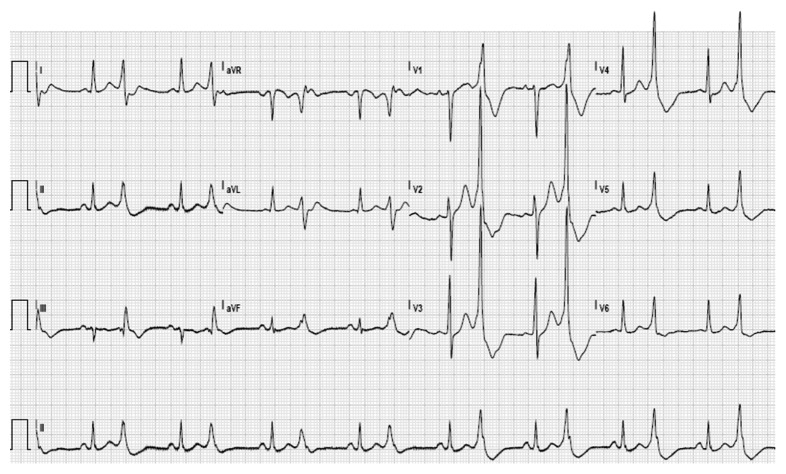
The 12-lead electrocardiogram with sinus rhythm (103 beats per minute) and frequent premature ventricular complexes in bigeminy pattern.

She was a nonsmoker, and there was no history of drug abuse. Current medications included diclofenac, mebeverine, and vitamin supplements. Her medical history consisted of a gastric banding operation one year ago, and prior to the event, she had only experienced some dizziness at times. The postoperative course of the gastric banding operation was uncomplicated. Her mother and four siblings lived in different countries around the world, so there was limited information about her family history. Her father passed away at the age of 55 due to complications of diabetes mellitus. The only thing that is known is that her family history was negative for SCD or cardiac disorders. Both of her sons had been diagnosed with autism but were in good physical health. The 12-lead electrocardiogram at presentation showed sinus rhythm with a frequency of 103 beats per minute and frequent premature ventricular complexes in bigeminy pattern with a QTc interval of 419 ms (Figure 1). A computed tomography (CT) scan of the thorax and cerebrum showed no evident abnormalities. After therapeutic hypothermia of 24 hours, she regained consciousness with reasonable neurological recovery without apparent sequela. Furthermore, a transthoracic echocardiogram demonstrated a good left and right ventricular function and no valvular abnormalities.

**Figure 2:**
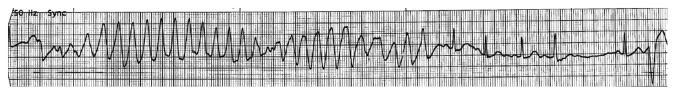
Rhythm strips with episodes of torsade des pointes terminating spontaneously.

**Figure 3:**
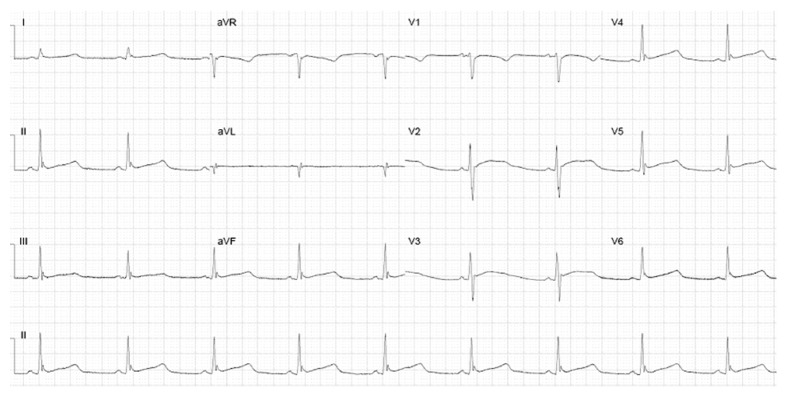
The 12-lead electrocardiogram after two days of hospitalization with a prolonged QTc interval of 526 ms.

Days after detubation, episodes of frequent premature beats, nonsustained ventricular tachycardias, and prolonged QTc intervals manifested, and treatment with propranolol was started. Furthermore, frequent episodes of torsade des pointes were observed (Figure 2). Cordarone was given, which resulted in progression of the torsade des pointes into VF requiring electrical cardioversion. Cordarone treatment was discontinued. Laboratory tests showed a mild hyponatremia of 134 mmol/l, a hypokalemia of 3.1 mmol/l, and a normal calcium level of 2.38 mmol/l. However, after electrolyte corrections, ventricular tachyarrhythmias persisted. During hospitalization, the QTc interval varied between normal (<460 ms) and severely prolonged (552 ms) (Figure 3). Due to bradycardias, treatment with propranolol was discontinued. Eventually, sinus rhythm was achieved with flecainide. Coronary angiography showed no coronary artery stenosis, and cardiac magnetic resonance imaging also revealed no abnormalities.

A dual-chamber implantable cardioverter-defibrillator was implanted for secondary prevention. Subsequently, genetic testing for the LQT1, LQT2, LQT3, LQT5, and LQT6 genes identified a heterozygous c.3092_3096dup (p.Arg1033ValfsX26) mutation of the *KCNH2* gene (LQT2) and a heterozygous c.170T > C (p.Ile57Thr) unclassified variant (UV) of the *KCNE2* gene (LQT6). The UV (missense mutation) of the *KCNE2* gene is likely a pathogenic mutation, what results in the digenic inheritance of LQT2 and LQT6. Genetic screening revealed that both sons are not carrying the familial *KCNH2* mutation. They have not been tested for the UV of the *KCNE2* gene.

## 3. Discussion

This case report describes a patient with an OHCA due to VF with the coexistence of a mutation in the *KCNH2* gene (LQT2) and an UV of the *KCNE2* gene (LQT6). An UV is a genetic sequence whose association with, in this case, LQTS is uncertain. In uncertain negative results, these UVs have not been found in affected counselees at high risk of LQTS. This is presumably the reason why both sons have not been tested for the UV of the *KCNE2* gene. Prior studies reported that this *KCNE2* UV is most likely a pathogenic mutation because it was not present in 1300 healthy controls, it causes spontaneous and drug-induced prolongation of cardiac repolarization, and it has a loss-of-function effect on the *I*_kr_ current [3, 5–7]. The *KCNH2* and *KCNE2* genes both encode the *I*_kr_ channel, but for different subunits. The *KCNH2* gene encodes the HERG subunits and the *KCNE2* gene the MiRP1 subunit. It was found that the MiRP1 mutants and the HERG mutants both form channels that conduct diminished potassium currents [5–8]. These data indicate that these two mutations combined might have an aggravating effect on loss of function of the *I*_kr_ channel.

Patients with digenic mutations are relatively common. Studies showed that in 4.6% to approximately 10.0% of the LQTS patients, two mutations were found [4, 9, 10]. To our knowledge, the coexistence of mutations in the LQT2 and LQT6 has never been described before. Mutations in the *KCNH2* gene are common and often involved in the digenic inheritance of LQTS. However, LQTS is in <1% caused by mutations in the *KCNE2* gene: Tester et al. found only one *KCNE2* mutation in 541 unrelated patients [4]. The coexistence of LQT2 and LQT6 in one individual is thus extremely rare.

Patients with two or more mutations were more likely to have a severe clinical manifestation of LQTS. The QTc intervals were longer, and patients had more often cardiac arrhythmias and symptoms such as syncope and cardiac arrest [9, 10]. Furthermore, the onset of cardiac events in digenic inheritance is at a significantly younger age (10 ± 8 years vs 18 ± 16 years) [11]. However, our patient was 37 years old when she experienced her first cardiac event. The late presentation could be related to the fact that this *KCNE2* UV in an isolated setting has not previously been associated with severe clinical manifestations, only a prolonged QT duration [12, 13]. A severe phenotype of this UV has only been described in combination with LQT3 in patients presenting with neonatal seizures and ventricular tachycardia [13]. Perhaps, exacerbation of symptoms for this *KCNE2* UV is solely triggered in susceptible situations, for example, use of drugs that inhibit cardiac potassium channels [6]. In digenic inheritance, the variant may therefore have a subordinate role and cause no aggravating phenotypic expression over the single LQTS mutation. The findings of Westenskow et al. and Schwartz et al. are based on other combinations of digenic mutations, and the clinical expression of the combination of LQT2 and LQT6 is not yet described and may differ. In addition, the disease course of this patient can also be attributed to the variable penetrance of LQTS. She had, however, experienced dizziness prior to the event, which may indicate she had unconsciously suffered from TdP.

Nevertheless, it is possible that the combination of LQT2/LQT6 occurs more frequently than reported. Because most genotype-positive patients have a mutation in *KCNQ1*, *KCNH2*, or *SCNA5* genes, it was recommended only to test these genes [14, 15]. However, in our case, mutations in other LQT genes could have been missed. Consequently, a potential (second) mutation will be left undiagnosed in the patient and family members. Because of the relatively high prevalence and predictive value of digenic mutations, screening of all the LQTS genes should be considered in every patient. As a result, there will be an increased risk of finding UVs which may give distress because of its uncertainty. However, if a patient carries a heterozygous mutation in two different genes, the risk that a child inherits both mutations is 25%, which implicates a more severe phenotype requiring close follow-up. By not screening all the LQTS genes, this would be missed. Napolitano et al. showed that, contrary to common perception, at least 88% of the LQTS patients have inherited the disease [14]. This states the importance of genetic screening of family members of LQTS patients as well. Furthermore, genetic screening will provide more information about the clinical presentation and incidence of the coexistence of LQT2 and LQT6.

## 4. Conclusion

The coexistence of long QT syndrome type 2 (*KCNH2* gene) and type 6 (*KCNE2* gene) is extremely rare. Screening of all the long QT syndrome genes should be considered in every patient and family member to not leave a potential (second) mutation undiagnosed.
